# The Necessity of Methodological Advances in Pain Research: Challenges and Opportunities

**DOI:** 10.3389/fpain.2021.634041

**Published:** 2021-05-26

**Authors:** Apkar Vania Apkarian

**Affiliations:** ^1^Department of Physiology, Anesthesiology, Physical Medicine and Rehabilitation, Northwestern University, Chicago, IL, United States; ^2^Center for Translational Pain Research, Center of Excellence for Chronic Pain and Drug Abuse Research, Feinberg School of Medicine, Northwestern University, Chicago, IL, United States

**Keywords:** methods, models, perception, opportunities, challenges

Innovation in methodology is fundamental to the advancement of science. Dissemination of such information, specifically in the field of pain research, has remained unstructured and distributed across various journals and monographs. It is thus imperative to create an openly shared space where descriptions of novel methods, specifically dedicated to the field of pain research, can be summarized.

## From Models to Methods: A Large-Brush Overview of Progress in the Field

There is little question that the mechanistic view of pain perception begins with Descartes' child in pain drawing from 1,644. This drawing introduced the concept of a sensory system, with components including peripheral encoding, afferent and central transmission, and representation/encoding/perceiving at the level of the cortex. Thus, the idea preconceived the last 150 years of research in sensory neuroscience. As such, to this day, Descartes' sketch remains a favorite and inspiring concept. Descartes deserves credit for formalizing the fundamental constituent components of the general idea of a sensory system via his illustration of the biological response to burning the skin (Although, there is good evidence that he may have borrowed this idea from Arab scientists). In a sense, Descartes' drawing summarized all of the modern neuroscience of sensation—it served as the initial grand model of how biology encodes sensation. Thus, it is a beautiful example of a paper-and-pencil cartoon model that sparked centuries of neuroscientific discoveries.

Moving forward to the late nineteenth century, German psychophysicists [Weber (1), Fechner (2), and others] initiated the debate as to whether sensations are quantifiable. The effort was a search for mathematical transformations between stimuli and the mind. This research created rules, tools, and methods at a time where statistical methods were still nascent. Their quantitative measurements began identifying various sources of variability: within a given subject, between subjects, and due to uncontrollable influences. In turn, the work paved the way for the discovery of the power law by Stevens (3), where the statistics of the stimulus-mind relationship for pain were shown to have the unique property of sensitization (power law with exponent > 1.0). This distinguished pain from all other sensations and mathematically demonstrated its necessity in escape and protection behaviors. These early psychophysics concepts formed the foundation of the modern pain perception scales now universally used in clinical trials (VAS, NRS); such subjective reports are now a staple across all of the pain management and treatment literature.

If one considers that the twentieth century established static characterization of pain, the new century paves the way for studying pain as a time varying dynamical system. The advent of the digital age facilitates the quantification of human pain perception at greater temporal resolutions, with which the dynamics of pain can be studied. For example, smartphone technology enables convenient, ecologically valid data collection at much greater sampling rates (4). Over shorter timescales, both acute and chronic pain have recently been examined from a dynamical viewpoint. For instance, different chronic pain conditions possess distinct fractal properties regarding spontaneous fluctuations (5). Similarly, high sampling rates have led to the discovery of “offset analgesia” (6), a contrast enhancement phenomenon that can be mathematically described using a second-order differential equation (7, 8). The latter is the only differential equation that can be related to any sensation. Thus, the sensory modality—pain—that has been philosophically expounded for centuries as the most incommunicable seems to be the one that can be precisely captured mathematically. The biological underpinnings of the statistical and dynamical properties of pain remain (for the most part) to be discovered. Such discoveries would undoubtedly provide novel mechanistic insights.

The 200-year history of advancements in unraveling pain magnitude perception has proven to be critical in clinical studies and applications. Notwithstanding, pain magnitude is also commonly acknowledged to be a very poor metric of both acute and chronic pain qualia. The latter has been supplemented with a long list of patient-reported outcomes (PROs), in which the properties, characteristics, and related psychological disturbances can be assessed in subjects with pain. Most notably, the McGill Pain Questionnaire (9) has been used extensively for this purpose. Perhaps surprisingly, the interrelationship between PROs and pain ratings, especially in the clinical setting, remains minimally explored. Early work within this purview demonstrates surprising relationships between personality, pain ratings, and clinical pain states (10). How such relationships evolve and differentiate between clinical conditions remains unexplored. Exploring these relationships necessitates much larger datasets than are typically collected in clinical pain research studies, but such efforts will undoubtedly be fruitful.

Conceptual models in pain research remain scarce. Melzack and Wall's gate control theory (11) is perhaps the most influential two-synapse model. It summarizes the concept of a specific spinal cord circuit to control ascending nociception. It is the basis for 50 years of follow-up research and the creation of many technologies for managing clinical pain; *viz*. electrical stimulators applied to different parts of the afferent-spinal circuitry. The model is a prime example of a concept leading to novel methods both for research and for clinical treatment. More recent conceptual models of the spinal circuitry are far more complex as they incorporate knowledge that has been amassed. Depending on one's question, one may need to consider many different types of neurons, receptors, and neurotransmitters, and also include the interface between glia and neurons (12). This system is further complicated by its ability to reorganize in distinct ways in various rodent models of persistent pain, where diverse types of peripheral injuries lead to distinct peripheral-spinal cord plasticity (13, 14). The cellular molecular details of nociceptive afferent inputs and their interaction in the spinal are now characterized (and continue to be investigated) at exquisite detail. The intent of this effort was to cure acute and chronic pain, yet we remain far from achieving these goals.

There is little doubt that the creation of alternate organism models of clinical pain states should be regarded as a major advance in pain research over the last 50 years. The discovery of a partial peripheral nerve injury in rodents giving rise to behaviors that correspond to human signs of persistent/chronic pain was an exciting observation that hugely influenced the field. The first robust model of this type was described by Bennett (15). This model (CCI) and its various variants continue to reveal important knowledge regarding the underlying mechanisms of chronic pain (16). The translational value of such models has also been criticized (16). Contrary to the initial enthusiasm, they have not resulted in the discovery of efficient treatments. Yet, their utility in advancing knowledge, especially regarding the plasticity of the peripheral and central neural circuits, cannot be questioned. More recent rodent models, capturing more specific properties of different human chronic clinical pain conditions, remain ongoing [e.g., see (17–19)]. It is also important to highlight the applicability of these rodent models in uncovering supraspinal neocortical and limbic brain circuitry in pain (20–22), even though these models were initially designed to capture peripheral-spinal cord interactions. One must then assume that such models will continue to generate new insights and ultimately lead to novel therapies for pain.

Aside from choosing appropriate models representing human clinical pain conditions (23), limitations in assessing pain in such models remain an important challenge. Assessing touch-induced withdrawal is prevailingly used as if it is a golden standard, but it is doubtful that this defensive response represents what is defined as “unpleasant sensory and emotional experience” in human subjects. Many different routes have been explored to expand on pain evaluation in model organisms. A broad range of behavioral measurements including the evaluation of social interactions, place preference/avoidance, elevated-plus maze, burrowing etc. have been explored, yet these efforts remain limited to specific studies or labs. Large data modeling approaches are beginning to make inroads in the topic as well. Computational technology allows for more ecological measurements of animal pain behaviors; tools from dynamical systems, statistical modeling, and machine learning enables the analysis of high dimensional behavioral data (e.g., hours of video recording) (24), or analyzing high-speed videos of paw movements (25).

Human brain imaging technology has advanced our notions of the brain circuitry implicated in pain and the discovery of biomarkers of risk for chronic pain. A combination of human brain imaging and rodent models has yielded a four-phase model of transition from acute to chronic pain (26, 27). The long-term influence of this model remains to be seen. However, it has already led to novel approaches in designing clinical trials for testing the efficacy of a combination medication therapy to block the transition from acute to chronic pain (28). Is remains unclear whether comparative brain imaging studies, between models and human conditions, would be an appropriate route with which similarity of brain activity patterns can be used to establish model correspondences to specific clinical conditions.

Nocifensive behavior is ubiquitous in living organisms, starting from unicellular life. Thus, it is unquestionable that nociceptive signaling, and related pain, are fundamental for survival and evolution. Surprisingly, little is known regarding the mechanisms and function of pain from an evolutionary context. The comparison of pain-related behaviors across species can help us in the distinction of pain and nociception (29), since both vertebrates and invertebrate animals share a similar segregation of nociceptive and non-nociceptive signals (30). However, quantifying pain remains a most complex task, especially because the current definition of pain– the conscious and emotional experience of pain—is human focused and defining conscious experiences outside of reported subjectivity remains unreachable (31, 32). Despite challenges, advances on the field show how pain-like behaviors interact with motivational effects as an adaptative response to nociceptive sensitization—which can be key for survival in some species (33); these motivational effects share similarities, in humans, with fear and anxiety derived pain behaviors which may become maladaptive (34). Experimental research can take in consideration predatory and defense interactions—in presence and absence of pain/injury—to further understand mechanism of hypervigilance and hyperalgesia expressed in mammals species. One could even argue that the ultimate nocifensive behavior is the establishment of culture: constructing shelter, moving into caves, and ultimately building cathedrals are seem evolutionarily aimed at enhancing comfort and thus also reducing nociception within the environment.

Models—whether they be toys (paper planes), circuit diagrams, mathematical formulations, or more general formulations (labeled line vs. distributed representation; the necessity of peripheral vs. central mechanisms)—remain essential to furthering science. Moreover, they are critical to conceptualizing and advancing and/or applying novel tools and methods. For a slightly different and more detailed outline of the current theories of mechanisms of pain, see (35). Importantly, the utility of models becomes most apparent when they are simplified to contain only their essential components. The latter is always a challenge for the biologist who is excited in continuously unraveling the huge complexity of the cellular and molecular construction of life.

## Challenges

In the field of clinical pain, the principal grand challenge remains the need to conquer chronic pain. The prevalence of chronic pain is on the rise; its ubiquity and healthcare cost worldwide is staggering. Somewhere between 15 and 20% of the world population suffers from continued, unremitting pain, for which there are no scientifically validated treatments. Therefore, new approaches in research methods and their application will be critical to advance our knowledge and build validated preventive and treatment methods. Such methods will likely need to be multi-modal; in this sense, proper co-implementation will be necessary to move the field forward. For further discussion on the challenges regarding the transition from acute to chronic pain, see (36); musculoskeletal pain, see (37); neuropathic pain, see (38); and challenges in the pharmacotherapy of pain, see (36). These latter articles outline the general scope and vision of Frontiers in Pain Research.

Of course, another major challenge is the scientific conceptualization of pain perception. Its definition at a brain representational level, at least for acute painful stimuli in healthy subjects, should have been settled years ago given the plethora of human brain imaging data. Yet, the topic remains hotly debated. For example, compare the strong claims of a whole-brain signature for acute pain (39) with the opposing view that the latter is not tenable (40, 41). It used to be thought that the sensory component of acute pain was represented in some differential pattern within S1 or S2 or insula, while its affective properties were localized to ACC (42). This is despite the ascending supraspinal nociceptive signals reaching the cortex have long been known to be minimal (43). There is now even evidence that one can capture signals related to acute pain from the primary visual cortex (44). Thus, even the existence of brain activity patterns that are specific to acute pain remains unclear. In fact, whether a brain pattern or interaction across many brain regions constructively defines pain states remains unsettled, and the challenge of isolating pain from tightly correlated mental states like salience, anxiety, and attention is also unsettled (45). Human studies and rodent models on the transition to chronic pain have vastly expanded our knowledge of the brain circuitry engaged in the process (27). In particular, by describing the critical role of mesolimbic motivational/affective circuits in the transition to chronic pain (46) and identifying predictive biomarkers for the development of chronic pain (26, 47). Current evidence on the mechanisms of plasticity, as well as risk predictive markers, suggests that these mechanisms may be critically dependent on the type of chronic pain. However, such knowledge remains to be systematically pursued. Thus, the minimal biological requirements for pain perception remain elusive. Similarly, the expansion of such circuits with distinct chronic pain conditions also remains unclear.

## Opportunities

In the twenty-first century, advances in biological science research methods continue to occur at a dizzying pace. Such advances span all domains and scales; from novel genetic tools to genetically modified rodent models, to cellular molecular techniques coupled with genetic tools to modulate neural circuitry, to new advances in clinical trial design that may be coupled with brain imaging and molecular and genetic assays. Various combinations of these methods should provide unprecedented opportunities to demystify acute and chronic pain. Yet, tools alone are not sufficient; they need to be coupled with models that can be rigorously tested.

Within the framework of preventing/curing chronic pain, there remain a multiplicity of interrelated challenges that require new approaches, combinations of approaches, and concepts (48). An important example is recent efforts to modify the definitions of pain and chronic pain (49). The latter is a natural consequence of advances in mechanistic understandings, which will continue to evolve as critical neural, glial, and genetic factors become more apparent. Finally, conflating pain and nociception emanates from the ongoing, opposing viewpoints regarding the necessary and sufficient neural elements that underscore pain and chronic pain, especially the supposedly opposing positions of peripheral vs. central circuitry being viewed as the prime contributors and thus controllers of pain states (50).

The persistence of the Covid19 pandemic itself poses additional challenges. Societal changes in everyday behaviors, more seclusion, and less physical interaction will further exacerbate the suffering of living with pain. Indeed, new data indicate that the opioid epidemic, a simple barometer of incidence and prevalence of chronic pain, is itself becoming more prevalent.

Human brain imaging methods have already revolutionized our concepts of pain and chronic pain. By themselves, these methods can only push the field so far. They need to be coupled with forward and backward translational approaches, along with causal studies of neuronal circuits by taking advantage of optogenetic and chemogenetic tools.

Decades of intense and costly research by the pharmaceutical industry have yielded very meager results. Progress in pharmacotherapy would need far better-combined studies of brain physiology, human genetics, and human behavior assessments, ideally tightly coupled with each other, and with translational animal model studies.

I hope and expect that this Methodology dedicated section of the journal can provide a platform for further expound, argue, and demonstrate the merits of the application of diverse methods to advancing the science of pain.

A major issue in advancing the field regards the rigor with which science is conducted. It is now apparent that more transparency and a greater expounding of methods and results are critical for replication and thus the advancement of science. Proper power calculations, their reporting, and rigorous statistical models are also necessary for enhancing scientific research. Moreover, the age of big data is upon us, together with the necessity of collaboration across labs and research groups. Especially in genetics and human brain imaging, as well as their combination, will require appreciable collaborative efforts with coordinated and transparent methodologies. Fortunately, multiple such efforts have already launched or are starting (e.g., OPPERA, MAPP, BACPAC, A2CPS), which should quicken the pace of progress in revealing mechanisms and providing proper cures for chronic pain.

## Author Contributions

The author confirms being the sole contributor of this work and has approved it for publication.

## Conflict of Interest

The author declares that the research was conducted in the absence of any commercial or financial relationships that could be construed as a potential conflict of interest.
